# Urinary Profile of Endogenous Gamma-Hydroxybutyric Acid and its Biomarker Metabolites in Healthy Korean Females: Determination of Age-Dependent and Intra-Individual Variability and Identification of Metabolites Correlated With Gamma-Hydroxybutyric Acid

**DOI:** 10.3389/fphar.2022.853971

**Published:** 2022-04-13

**Authors:** Suji Kim, Suein Choi, Min Seo Lee, Mingyu Kim, Maria Park, Sungpil Han, Seunghoon Han, Hye Suk Lee, Sooyeun Lee

**Affiliations:** ^1^ Analytical Toxicology Laboratory, College of Pharmacy, Keimyung University, Daegu, South Korea; ^2^ Pharmacometrics Institute for Practical Education and Training, College of Medicine, The Catholic University of Korea, Seoul, South Korea; ^3^ Drug Metabolism and Bioanalysis Laboratory and BK21 Four-Sponsored Advanced Program for SmartPharma Leaders, College of Pharmacy, The Catholic University of Korea, Bucheon, South Korea

**Keywords:** gamma-hydroxybutyric acid, biomarker metabolites, urine, metabolite profiling, liquid chromatography-tandem mass spectrometry

## Abstract

Gamma-hydroxybutyric acid (GHB), used as a therapeutic and an illegal anesthetic, is a human neurotransmitter produced during gamma-aminobutyric acid (GABA) biosynthesis and metabolism. Potential biomarker metabolites of GHB intoxication have been identified previously; however, reference concentrations have not been set due to the lack of clinical study data. Urinary profiling of endogenous GHB and its biomarker metabolites in urine samples (*n* = 472) of 206 healthy females was performed based on differences in age and time of sample collection using liquid chromatography-tandem mass spectrometry following validation studies. The unadjusted and creatinine-adjusted urinary concentrations ranges were obtained after urinary profiling. The creatinine-adjusted concentrations of glutamic and succinic acids and succinylcarnitine significantly increased, whereas that of glycolic acid significantly decreased with advancing age. Significant inter-day variation of GABA concentration and intra-day variation of 3,4-dihydroxybutyric acid and succinylcarnitine concentrations were observed. The urinary concentrations of 2,4-dihydroxybutyric acid, succinic acid, and 3,4-dihydroxybutyric acid showed the highest correlation with that of GHB. Data from this study suggest population reference limits to facilitate clinical and forensic decisions related to GHB intoxication and could be useful for identification of biomarkers following comparison with urinary profiles of GHB-administered populations.

## Introduction

Gamma-hydroxybutyric acid (GHB) is an endogenous substance produced during the metabolism of gamma-aminobutyric acid (GABA), a major inhibitory neurotransmitter, in the human brain. GHB exerts sedative, hypnotic, and anesthetic effects by binding to the B sub-type of the GABA receptor and to a GHB-specific receptor in the central nervous system (CNS). It is also present in small amounts in natural food, such as some wines, meats, and fruits but exerts negligible effect on the human body upon ingestion. The sodium salt of GHB, sodium oxybate, used as a CNS depressant in the treatment of narcolepsy and alcoholism, is often misused in drug-facilitated crimes (Busardo and Jones, 2019; Trombley et al., 2019).

Numerous clinical and forensic studies on the distribution of endogenous GHB in human urine or blood have been performed to differentiate between endogenous and exogenous GHB in the body (Andresen et al., 2010; Busardo and Jones, 2019; Strickland et al., 2020). According to the reference range for urine samples, a cut-off value of 10 μg/ml GHB is generally accepted for forensic purposes; however, the analytical results in actual cases are at risk of false positive or false negative interpretation due to the *in vitro* production and rapid metabolism, with a half-life of 30–50 min, of GHB (Kang et al., 2014; Busardo and Jones, 2019). To circumvent these issues, metabolomics studies have been performed to identify alternative GHB intoxication markers (Jung et al., 2021). Urine metabolomics following GHB exposure have demonstrated that GHB administration to rats alters the metabolism of amino acids (Seo et al., 2018), polyamines (Lee et al., 2019), and organic acids related to the tricarboxylic acid (TCA) cycle (Seo et al., 2016b). Another study confirmed glycolic acid and succinic acid as potent urinary markers of GHB exposure (Palomino-Schatzlein et al., 2017). Further, the potential of metabolites such as 2,4-dihydroxybutyric acid (2,4-OH-BA) and 3,4-dihydroxybutyric acid (3,4-OH-BA) in phase 1 and GHB-glucuronide and GHB-sulfate in phase 2, as alternative markers of GHB exposure, has been investigated (Jarsiah et al., 2020; Jung et al., 2021). Moreover, new urinary GHB biomarkers, including succinylcarnitine and GHB-conjugates (GHB-carnitine, GHB-glutamate, and GHB-glycine), have been identified (Steuer et al., 2019).

**FIGURE 1:**
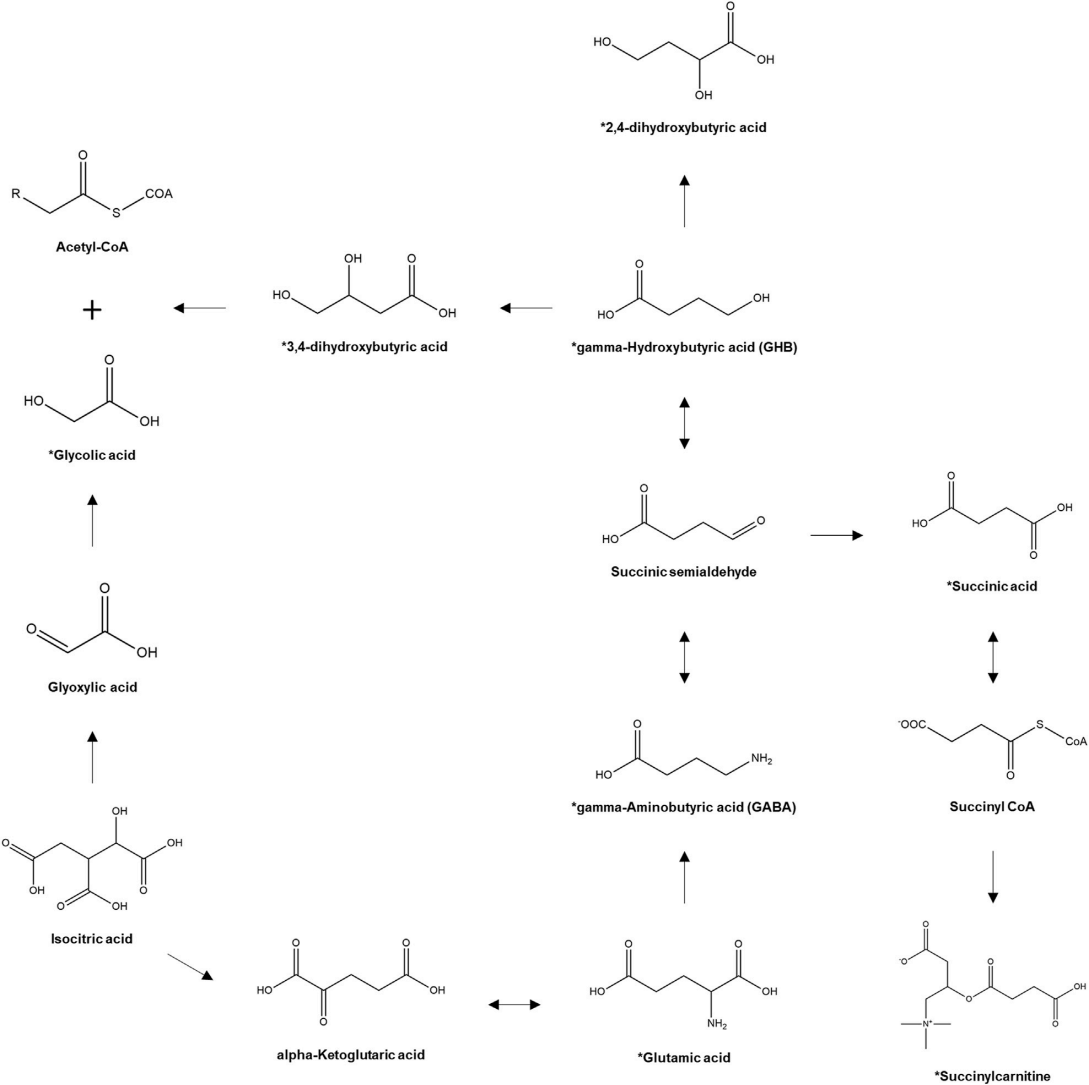
Pathway of GHB biosynthesis and metabolism. *, target analytes. (Kim et al., 2022).

However, despite the identification of potential diagnostic biomarkers of GHB intoxication, GHB continues to be used primarily in clinical and forensic settings. One of the main reasons for this is that the reference concentrations of the potential biomarkers, which are also endogenous metabolites, have not been standardized yet. Moreover, intra-individual variations that depend on circadian rhythm or diet and inter-individual variations, such as gender and age, possibly affect the concentrations of the endogenous metabolites (Slupsky et al., 2007; Bertram et al., 2009; Dallmann et al., 2012; van Duynhoven and Jacobs, 2016). Therefore, the feasibility of the potential biomarker metabolites of GHB exposure needs to be examined keeping the inter- and intra-individual variations under consideration. In this study, we selected the most promising biomarker metabolites of GHB exposure (glutamic acid, GABA, succinic acid, 2,4-OH-BA, 3,4-OH-BA, glycolic acid, and succinylcarnitine) [Figure 1, (Kim et al., 2022)] based on the results of previous metabolic studies (Jarsiah et al., 2020; Jung et al., 2021) and investigated their urinary profiles in 206 healthy adult Korean females. The concentrations of endogenous GHB and biomarker metabolites in 472 urine samples collected from these females at various time points were quantified using fully validated liquid chromatography-tandem mass spectrometry (LC-MS/MS) followed by comparisons based on age and day and time of sample collection. Furthermore, metabolites having a high correlation with endogenous urinary GHB were identified.

## Materials and Methods

### Clinical Study Subjects

This study was approved by the ethics committee of Seoul St. Mary’s Hospital of the Catholic University of Korea (Seoul, Republic of Korea, approval number: KC20TISI0193, approval date: 08 April 2020). All subjects provided informed consent prior to enrollment in the clinical study. The subjects comprised healthy Korean females, aged 20–49 years, weighing 40 kg or above but within ±20% of the ideal body weight. Subjects having any history of clinically significant cardiovascular, respiratory, renal, endocrine, hematological, digestive, central nervous system, and psychiatric diseases, malignant tumors, and/or drug abuse or those who were on medication that could affect the evaluation of endogenous GHB and related metabolites were excluded from the study.

### Urine Sampling and General Urine Analysis

Selected subjects from the Seoul St. Mary’s Hospital were recruited in two study groups: inter-day and intra-day. Subjects in the inter-day study were asked to visit the hospital on two different days of the week and provide a spot urine sample on each day, whereas those in the intra-day study were hospitalized for 2 days and asked to provide spot urine samples during the hours of 18:00–23:00 on the first day and 4:00–10:00, 10:00–14:00, and 14:00–18:00 on the second day. All urine samples were immediately submitted to the Department of Laboratory Medicine for general urine analysis. Creatinine levels were measured using the Clinical Analyzer 7600 Series (HITACHI, Tokyo, Japan) using compensated rate-blanked Jaffe kinetic assay. The levels of glucose, leukocyte, bilirubin, ketone, protein, urobilinogen, and nitrite and urine color and pH were determined via a single dipstick urinalysis using URiSCAN 11 strip (YD Diagnostics, Gyeonggi-do, South Korea). The results of the dipstick urinalysis (except for urine color and pH) were interpreted according to a color scale: negative, trace, 1+, 2+, 3+, or 4+, using URiSCAN Super+ (YD Diagnostics). The urine samples were stored at −70°C until LC-MS/MS analysis.

### Urinary Profiling of Endogenous Gamma-Hydroxybutyric Acid and its Biomarker Metabolites

Chemicals and reagents used for urinary profiling of endogenous GHB and its biomarker metabolites are listed in Supplementary Method S1. Urinary profiling of endogenous GHB and its biomarker metabolites was performed using two distinct LC-MS/MS methods: method 1 (without benzoylation) (Supplementary Methods S2) was utilized to profile GHB, whereas method 2 (with benzoylation) (Supplementary Methods S3) was utilized to profile other metabolites. In case of both methods, LC-MS/MS analyses were conducted using a 1290 infinity LC system (Agilent Technologies, Santa Clara, CA, United States) and 6495 triple quadrupole MS/MS (Agilent Technologies). Data were processed using the MassHunter software (B. 07. 01, Agilent Technologies). Both methods were fully validated, as described in Supplemental Method S4.

### Statistical Analysis

To compare the demographics and urinalysis results among the age groups, a one-way analysis of variance (ANOVA) and Pearson’s Chi-squared test were performed for continuous and categorical variables, respectively, using R (version 4.1.0). Statistical analysis of creatinine and the unadjusted or creatinine-adjusted concentrations of GHB and its metabolites was performed using GraphPad Prism 8 (version 8.0.2, GraphPad Software, La Jolla, CA, United States). Indications of significance were based on results obtained from Tukey’s *post-test* following a one-way ANOVA for comparison among more than three groups and paired *t*-test for that between two groups. Pearson correlation coefficients were applied to the unadjusted and creatinine-adjusted concentrations of GHB and metabolites to identify metabolites correlated with GHB.

## Results

### Quantification of Endogenous Gamma-hydroxybutyric acid and its Biomarker Metabolites in the Urine of Healthy Korean Females

In total, 206 healthy female subjects completed the inter-day (Age: 20–29 years (20s), *n* = 58; Age: 30–39 years (30s), *n* = 59; Age 40–49 years (40s), *n* = 59) and intra-day study (20s, *n* = 10; 30s, *n* = 10; 40s, *n* = 10). All subjects were Korean (East Asian ethnic group) with comparable demographics. The results of the clinical urinalysis that include the levels of glucose, leukocyte, bilirubin, ketone, protein, urobilinogen, and nitrite and urine color and pH are summarized in Table 1. These parameters were generally within the normal range; no statistically significant differences were observed among the age groups. Dark red urine was observed in two participants, and they were included in the study because no pathological conditions and specifics were found in the questionnaire and medical history. GHB and its biomarker metabolites in the urine samples were quantified using fully validated methods, the results of which are presented in Supplementary Results S1, Supplementary Figure S1, Supplementary Table S2.

**TABLE 1:** Demographics and clinical urinalysis of subjects according to age groups in the inter- and intra-day studies.

Group		Inter-day study			*p* value	Intra-day study			*p* value
Age		20s	30s	40s	-	20s	30s	40s	-
Number of subjects		58	59	59	-	10	10	10	-
Height (cm, mean ± SD)		162.5 ± 5.1	161.4 ± 4.8	160.5 ± 5.6	0.122	164.9 ± 4.4	160.2 ± 6.4	161.0 ± 4.8	0.119
Body weight (kg, mean ± SD)		55.6 ± 7.3	55.7 ± 6.5	58.5 ± 7.4	0.039	54.2 ± 3.5	56.4 ± 6.1	57.9 ± 6.0	0.322
Number of samples		116	118	118	-	40	40	40	-
Color [n, (%)]	Dark red	1 (0.9)	1 (0.8)	0 (0.0)	0.378	0 (0.0)	0 (0.0)	0 (0.0)	0.549
	Light yellow	109 (94.0)	108 (91.5)	115 (97.5)		40 (100.0)	39 (97.5)	38 (95.0%)	
	Orange	0 (0.0)	0 (0.0)	0 (0.0)		0 (0.0)	0 (0.0)	1 (2.5)	
	Yellow	6 (5.2)	9 (7.6)	3 (2.5)		0 (0.0)	1 (2.5)	1 (2.5)	
pH (mean ± SD)		6.1 ± 0.5	6.1 ± 0.6	6.2 ± 0.6	0.168	6.1 ± 0.6	6.2 ± 0.6	6.1 ± 0.7	0.824
Glucose [n, (%)]	Negative	116 (100.0)	118 (100.0)	116 (98.3)	0.136	40 (100.0)	40 (100.0)	39 (97.5)	0.365
	Trace	0 (0.0)	0 (0.0)	2 (1.7)		0 (0.0)	0 (0.0)	1 (2.5)	
Leukocyte [n, (%)]	Negative	55 (47.4)	73 (61.9)	73 (61.9)	0.142	32 (80.0)	27 (67.5)	36 (90.0)	0.232
	Trace	27 (23.3)	26 (22.0)	18 (15.3)		4 (10.0)	8 (20.0)	3 (7.5)	
	1+	19 (16.4)	11 (9.3)	13 (11.0)		4 (10.0)	4 (10.0)	1 (2.5)	
	2+	12 (10.3)	6 (5.1)	8 (6.8)		0 (0.0)	0 (0.0)	0 (0.0)	
	3+	3 (2.6)	2 (1.7)	6 (5.1)		0 (0.0)	1 (2.5)	0 (0.0)	
Bilirubin [n, (%)]	Negative	116 (100.0)	118 (100.0)	117 (99.2)	0.370	40 (100.0)	40 (100.0)	40 (100.0)	-
	1+	0 (0.0)	0 (0.0)	1 (0.8)		0 (0.0)	0 (0.0)	0 (0.0)	
Ketone [n, (%)]	Negative	112 (96.6)	115 (97.5)	116 (98.3%)	0.206	40 (100.0)	35 (87.5)	39 (97.5%)	0.212
	Trace	2 (1.7)	1 (0.8)	2 (1.7)		0 (0.0)	2 (5.0)	1 (2.5)	
	1+	0 (0.0)	2 (1.7)	0 (0.0)		0 (0.0)	2 (5.0)	0 (0.0)	
	2+	2 (1.7)	0 (0.0)	0 (0.0)		0 (0.0)	1 (2.5)	0 (0.0)	
Protein [n, (%)]	Negative	110 (94.8)	110 (93.2)	114 (96.6)	0.756	37 (92.5)	39 (97.5)	36 (90.0)	0.741
	Trace	3 (2.6)	4 (3.4)	3 (2.5)		2 (5.0)	1 (2.5)	2 (5.0)	
	1+	3 (2.6)	3 (2.5)	1 (0.8)		1 (2.5)	0 (0.0)	1 (2.5)	
	2+	0 (0.0)	1 (0.8)	0 (0.0)		0 (0.0)	0 (0.0)	1 (2.5)	
Urobilinogen [n, (%)]	Trace	115 (99.1)	118 (100.0)	118 (100.0)	0.361	40 (100.0)	40 (100.0)	40 (100.0)	-
	1+	1 (0.9)	0 (0.0)	0 (0.0)		0 (0.0)	0 (0.0)	0 (0.0)	
Nitrite [n, (%)]	Negative	116 (100.0)	118 (100.0)	118 (100.0)	-	40 (100.0)	40 (100.0)	40 (100.0)	-

SD, standard deviation; 20s, 20–29 years; 30s, 30–39 years; 40s, 40–49 years.

**FIGURE 2:**
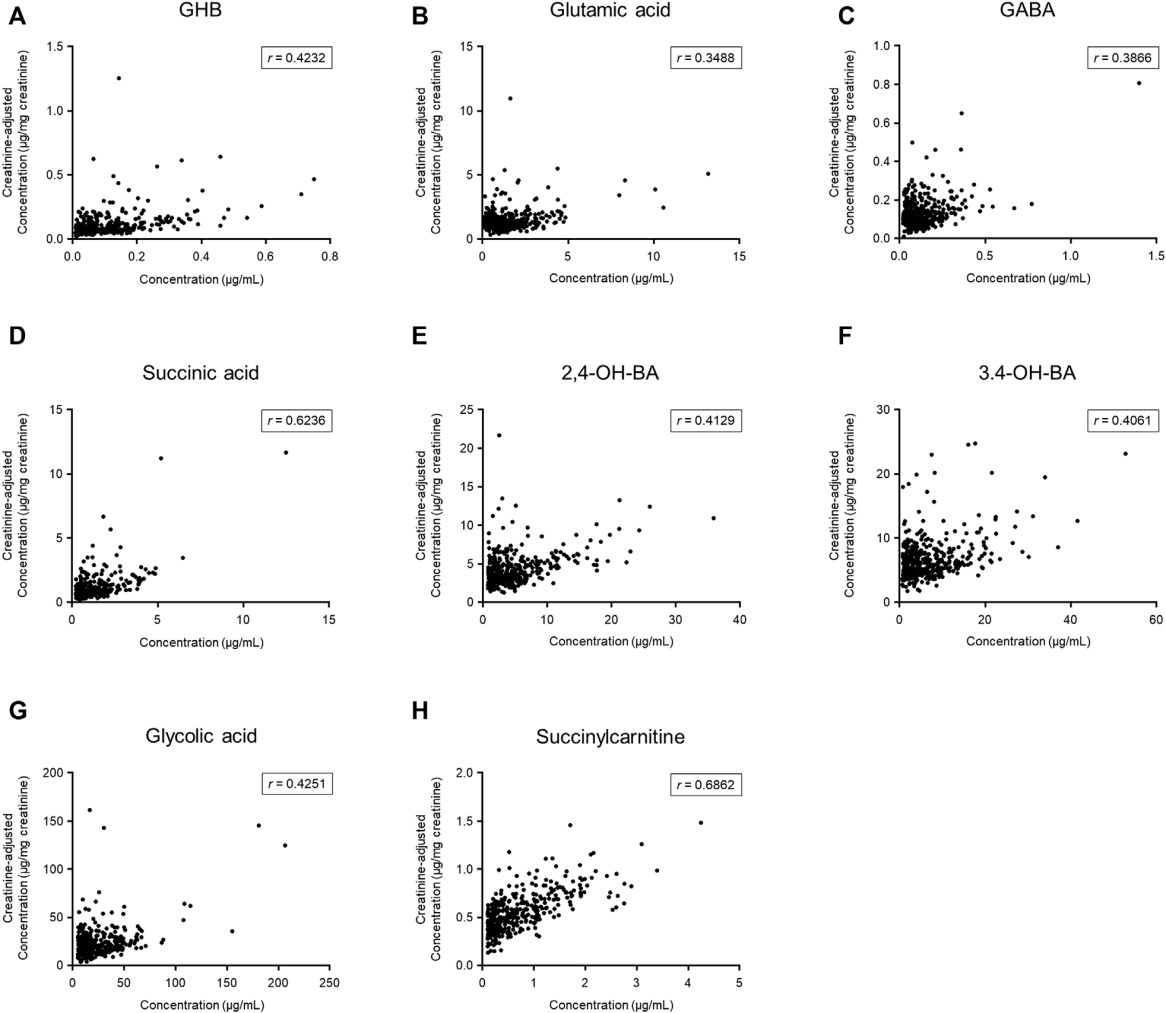
Comparison between unadjusted and creatinine-adjusted concentrations of gamma-hydroxybutyric acid (GHB) **(A)**, glutamic acid **(B)**, gamma-aminobutyric acid (GABA) **(C)**, succinic acid **(D)**, 2,4-dihydroxybutyric acid (2,4-OH-BA) **(E)**, 3,4-dihydroxybutyric acid (3,4-OH-BA) **(F)**, glycolic acid **(G)**, and succinylcarnitine **(H)** in urine samples (*n* = 472) of 206 subjects. Values less than the limit of quantification were excluded.

The numbers of urinary samples in which the concentration of the target compounds was higher than the lower limit of quantification were between 383 (succinylcarnitine) and 472 (glutamic acid); the quantitative results are summarized in Table 2. The creatinine-adjusted concentrations of the target compounds are also shown in Table 2. The creatinine-adjusted concentration of succinic acid (*r* = 0.6236) and succinylcarnitine (*r* = 0.6862) showed a significant correlation with their unadjusted concentrations, whereas that of the other compounds did not (GHB, *r* = 0.4232; glutamic acid, *r* = 0.3488; GABA, *r* = 0.3866; 2,4-OH-BA, *r* = 0.4129; 3,4-OH-BA, *r* = 0.4061; glycolic acid, *r* = 0.4251) (Figure 2).

**TABLE 2:** Unadjusted and creatinine-adjusted concentrations of endogenous GHB and its biomarker metabolites in urine samples (n = 472) of 206 adult Korean females.

Compound name		GHB	Glutamic acid	GABA	Succinic acid	2,4-OH-BA	3,4- OH-BA	Glycolic acid	Succinylcarnitine
Number of samples		460	471	439	386	429	466	404	383
Unadjusted concentrations (μg/ml)	Mean	0.10	1.33	0.13	1.15	4.61	6.67	25.5	0.72
	Median	0.07	0.92	0.10	0.74	3.15	4.53	17.7	0.51
	Range	0.01–0.75	0.11–13.2	0.02–1.40	0.20–12.5	0.78–35.9	0.66–52.8	5.07–207	0.1–4.25
	SD	0.10	1.37	0.12	1.17	4.59	6.52	26.8	0.65
Creatinine-adjusted concentrations (μg/mg creatinine)	Mean	0.10	1.41	0.14	1.08	4.26	6.62	24.6	0.55
	Median	0.08	1.25	0.12	0.82	3.73	6.08	20.3	0.52
	Range	0.02–1.25	0.33–11.0	0.01–0.81	0.21–11.7	1.26–21.7	1.8–24.8	3.51–162	0.13–1.48
	SD	0.10	0.84	0.08	1.07	2.13	3.14	20.7	0.21

The values less than the limit of quantification were excluded. SD, standard deviation; GHB, gamma-hydroxybutyric acid; GABA, gamma-aminobutyric acid; 2,4-OH-BA, 2,4-dihydroxybutyric acid; 3,4-OH-BA, 3,4-dihydroxybutyric acid.

### Age-Dependent Variabilities in Endogenous Concentrations of Gamma-Hydroxybutyric Acid and its Biomarker Metabolites

**FIGURE 3:**
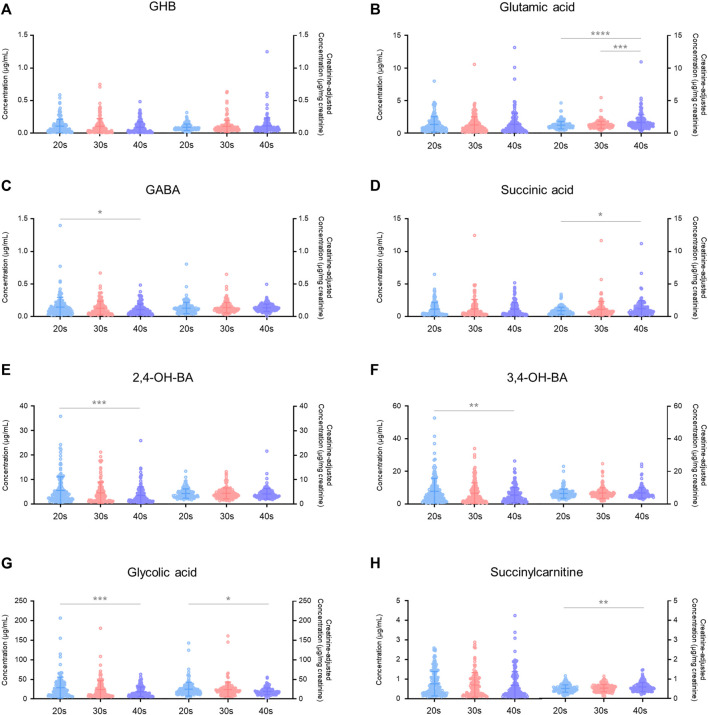
Age-dependent variabilities in unadjusted and creatinine-adjusted concentrations of GHB **(A)**, glutamic acid **(B)**, GABA **(C)**, succinic acid **(D)**, 2,4-OH-BA **(E)**, 3,4-OH-BA **(F)**, glycolic acid **(G)**, and succinylcarnitine **(H)** in 472 urine samples. Horizontal lines within the scattered dots indicate means ± standard deviations. Values less than the limit of quantification were excluded. *p < 0.05, **p < 0.01, ***p < 0.001, ****p < 0.0001.

Age-dependent variabilities in unadjusted and creatinine-adjusted concentrations of GHB and its biomarker metabolites in urine samples (*n* = 472) of the 206 subjects were analyzed (Figure 3). The quantitative results are summarized in Supplementary Table S3. The unadjusted concentrations of GABA (20s vs. 40s, *p* < 0.05), 2,4-OH-BA (20s vs. 40s, *p* < 0.001), 3,4-OH-BA (20s vs. 40s, *p* < 0.01), and glycolic acid (20s vs. 40s, *p* < 0.001) in the urine of subjects in their 20s were significantly higher as compared to those in the urine of subjects in their 40 s, whereas those of other metabolites were not significantly different between the age groups. The creatinine-adjusted concentrations of glutamic acid (20s vs. 40s, *p* < 0.001; 30s vs. 40s, *p* < 0.001), succinic acid (20s vs. 40s, *p* < 0.05), and succinylcarnitine (20s vs. 40s, *p* < 0.01) significantly increased, whereas that of glycolic acid (20s vs. 40s, *p* < 0.05) significantly decreased with advancing years. The creatinine concentrations in the urine samples varied significantly with age (20s vs. 40 s, *p* < 0.01, Table 3). Creatinine concentrations (mean ± SD) in urine samples of subjects in their 20s, 30s, and 40s were 117 ± 87 mg/dl, 101 ± 87 mg/dl, and 86 ± 70 mg/dl, respectively.

**TABLE 3:** Creatinine concentrations in urine samples.

	Age difference			Inter-day variability		Intra-day variability (hours)			
	20s	30s	40s	Day 1	Day 2	18:00–23:00	4:00–10:00	10:00–14:00	14:00–18:00
Number of samples	158	159	159	180	176	30	30	30	30
Mean (mg/dl)	117	101	86	105	105	126	135	64	40
Median (mg/dl)	95	69	63	71	82	114	116	40	28
Range (mg/dl)	9.6–437	8.5–427	4.7–343	4.7–437	8.9–427	8.5–343	37–328	10–207	10–167
Standard deviation (mg/dl)	87	87	70	90	80	78	74	53	32

20s, 20–29 years; 30s, 30–39 years; 40s, 40–49 years. 20 vs. 40s, *p* < 0.01; 18:00–23:00 vs. 10:00–14:00, *p* < 0.001; 18:00–23:00 vs. 14:00–18:00, *p* < 0.0001; 4:00–10:00 vs. 10:00–14:00, *p* < 0.001; 4:00–10:00 vs. 14:00–18:00, *p* < 0.0001.

### Intra-Individual Variabilities in Endogenous Concentrations of Gamma-Hydroxybutyric Acid and its Biomarker Metabolites

**FIGURE 4:**
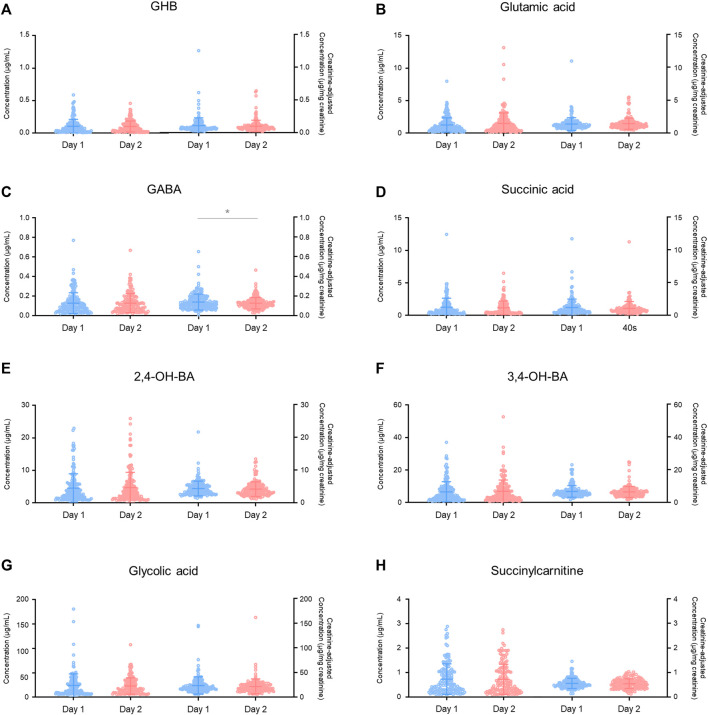
Inter-day variabilities in unadjusted and creatinine-adjusted concentrations of GHB **(A)**, glutamic acid **(B)**, GABA **(C)**, succinic acid **(D)**, 2,4-OH-BA **(E)**, 3,4-OH-BA **(F)**, glycolic acid **(G)**, and succinylcarnitine **(H)**. Horizontal lines within the scattered dots indicate means ± standard deviations. Values less than the limit of quantification were excluded. *p < 0.05.

**FIGURE 5:**
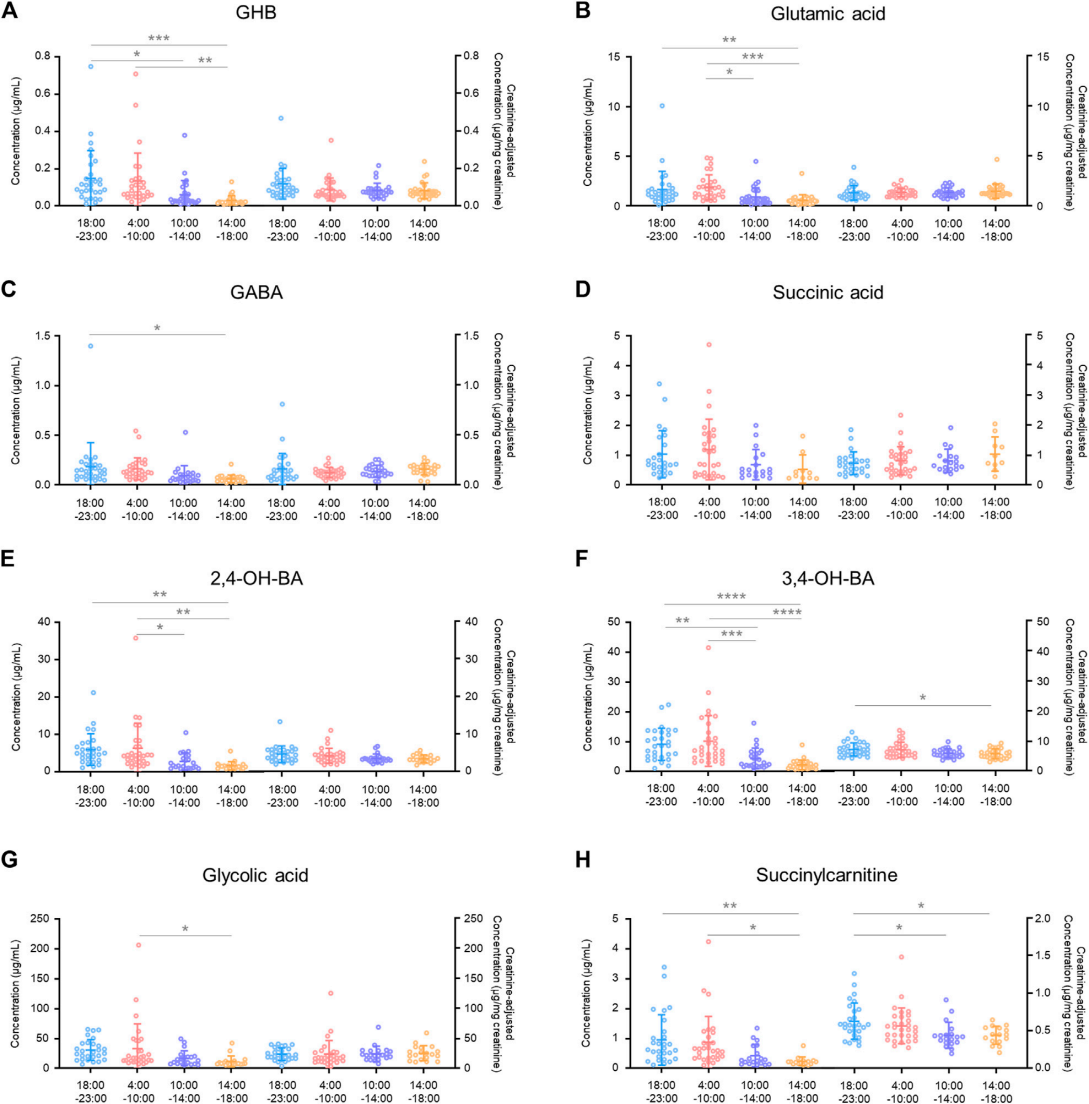
Intra-day variabilities in unadjusted and creatinine-adjusted concentrations of GHB **(A)**, glutamic acid **(B)**, GABA **(C)**, succinic acid **(D)**, 2,4-OH-BA **(E)**, 3,4-OH-BA **(F)**, glycolic acid **(G)**, and succinylcarnitine **(H)**. Horizontal lines within the scattered dots indicate means ± standard deviations. Values less than the limit of quantification were excluded. *p < 0.05, **p < 0.01, ***p < 0.001, ****p < 0.0001.

Inter-day (176 subjects) (Figure 4) and intra-day (30 subjects) (Figure 5) variabilities in unadjusted and creatinine-adjusted concentrations of GHB and its biomarker metabolites in urine samples were analyzed; the quantitative results are summarized in Supplementary Tables S3, S4, respectively. No significant difference was observed in the unadjusted and creatinine-adjusted concentrations of GHB and its biomarker metabolites between two different days, except in the creatinine-adjusted concentration of GABA (*p* < 0.05, Figure 4). There was no significant difference in the creatinine concentration (mean ± SD) between day 1 (105 ± 90 mg/dl) and day 2 (105 ± 80 mg/dl) (Table 3).

There was a significant difference in the creatinine-unadjusted concentrations of all the compounds, except for succinic acid, in the intra-day variability study, depending on the time of collection during a 24 h period (GHB, *p* < 0.001 in 18:00–23:00 vs. 14:00–18:00, *p* < 0.05 in 18:00–23:00 vs. 10:00–14:00, and *p* < 0.01 in 4:00–10:00 vs. 14:00–18:00; glutamic acid, *p* < 0.01 in 18:00–23:00 vs. 14:00–18:00, *p* < 0.001 in 4:00–10:00 vs. 14:00–18:00, *p* < 0.05 in 4:00–10:00 vs. 10:00–14:00; GABA, *p* < 0.05 in 18:00–23:00 vs. 14:00–18:00; 2,4-OH-BA, *p* < 0.01 in 18:00–23:00 vs. 14:00–18:00, *p* < 0.01 in 4:00–10:00 vs. 14:00–18:00, and *p* < 0.05 in 4:00–10:00 vs. 10:00–14:00; 3,4-OH-BA, *p* < 0.0001 in 18:00–23:00 vs. 14:00–18:00, *p* < 0.001 in 4:00–10:00 vs. 14:00–18:00, *p* < 0.01 in 18:00–23:00 vs. 10:00–14:00, and *p* < 0.001 in 4:00–10:00 vs. 10:00–14:00; glycolic acid, *p* < 0.05 in 4:00–10:00 vs. 10:00–14:00; succinylcarnitine, *p* < 0.01 in 18:00–23:00 vs. 14:00–18:00, and *p* < 0.05 in 4:00–10:00 vs. 14:00–18:00). However, after correction with creatinine concentrations, the deviation was reduced (3,4-OH-BA, *p* < 0.05 in 18:00–23:00 vs. 14:00–18:00; succinylcarnitine, *p* < 0.05 in 18:00–23:00 vs. 14:00–18:00, and *p* < 0.05 in 18:00–23:00 vs. 10:00–14:00) or eliminated (GHB, glutamic acid, GABA, 2,4-OH-BA, glycolic acid). The creatinine concentrations were significantly different depending on the time of collection during the day (18:00–23:00 vs. 10:00–14:00, *p* < 0.001; 18:00–23:00 vs. 14:00–18:00, *p* < 0.0001; 4:00–10:00 vs. 10:00–14:00, *p* < 0.001; 4:00–10:00 vs. 14:00–18:00, *p* < 0.0001) with the mean ± SD for 18:00–23:00, 4:00–10:00, 10:00–14:00, and 14:00–18:00 being 126 ± 78 mg/dl, 135 ± 74 mg/dl, 64 ± 53 mg/dl, 40 ± 32 mg/dl, respectively (Table 3).

### Identification of Biomarker Metabolites Correlated With Gamma-Hydroxybutyric Acid

**FIGURE 6:**
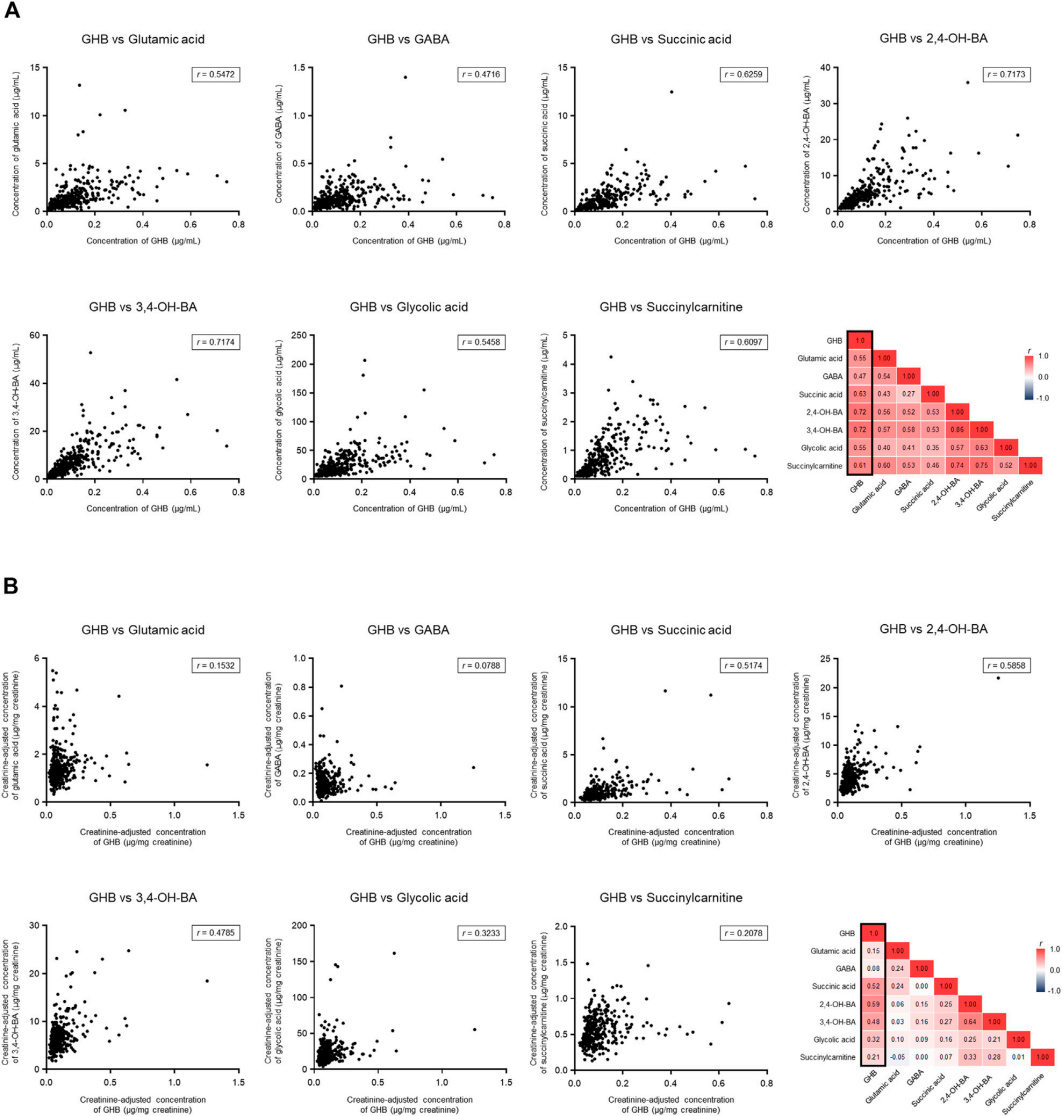
Correlation between endogenous GHB and its biomarker metabolites. **(A)** Unadjusted concentrations. **(B)** Creatinine-adjusted concentrations. Values less than the limit of quantification were excluded.

The creatinine-unadjusted concentrations of 2,4-OH-BA (*r* = 0.72), 3,4-OH-BA (*r* = 0.72), succinic acid (*r* = 0.63), and succinylcarnitine (*r* = 0.61) were highly correlated with that of GHB (Figure 6). Further, the creatinine-adjusted concentrations of 2,4-OH-BA (*r* = 0.59), succinic acid (*r* = 0.52), and 3,4-OH-BA (*r* = 0.48) showed the highest correlation with that of GHB (Figure 6).

## Discussion

Although previous studies have identified diagnostic biomarkers of GHB intoxication (Seo et al., 2016b; Palomino-Schatzlein et al., 2017; Seo et al., 2018; Lee et al., 2019; Steuer et al., 2019; Jarsiah et al., 2020; Jung et al., 2021), the use of metabolic precursors or products of GHB as alternative markers to differentiate between exogenous and endogenous GHB has had some limitations in clinical and forensic practice, primarily due to the lack of human data. In this study, 472 urine samples from 206 healthy females were analyzed to propose population reference concentrations to facilitate clinical and forensic decisions related to GHB intoxication. To the best of our knowledge, this is the first report of unadjusted and creatinine-adjusted concentrations of GHB and its biomarker metabolites in a significantly large number of human urine samples.

Wide variations have been reported in the endogenous concentrations of GHB in human urine in previous studies (Brailsford et al., 2010). In the present study, the maximum concentration of endogenous GHB detected in urine was 0.75 μg/ml, which is much lower than that previously reported (Brailsford et al., 2010). It is presumed that *in vitro* production during transport or improper storage conditions was the reason for this discrepancy. In previous studies on endogenous GHB concentration in urine, the samples were stored at −20°C (for a maximum of 36 months), 4°C, or 5°C (for a maximum of 33 days) (LeBeau et al., 2002; Elliott, 2003; Yeatman and Reid, 2003; Crookes et al., 2004; LeBeau et al., 2006; Brailsford et al., 2010). The urine samples in the current study were stored at −70°C immediately following collection and handled in an ice bath during the entire course of sample preparation, based on the results of the previous stability experiment (Kang et al., 2014). Few studies have been previously conducted on the endogenous urinary concentrations of biomarker metabolites of GHB. In a previous study on determining 10 urinary neurotransmitters and their metabolites in urine samples of six males and four females, glutamic acid and GABA levels were measured, and their concentrations were 3.050 ± 0.150 and 0.543 ± 0.036 μg/ml, respectively (Yan et al., 2017). Further, phase 1 metabolite and GHB levels were measured in urine samples of 77 males and 55 females with ranges as follows: GHB, not detected–1.94 μg/ml, glycolic acid, 1.30–400 μg/ml; succinic acid, 1.17–273 μg/ml; 2,4-OH-BA, 0.72–26.2 μg/ml; and 3,4-OH-BA, 1.88–122 μg/ml (Jarsiah et al., 2020). Most concentrations determined in the current study were lower than the previously reported maximum concentrations.

Creatinine adjustment of the concentration of drugs or metabolites in urine improves the reliability of the quantitative results as it compensates for the individual variations in the physiological dilution of urine (Lafolie et al., 1991). In a previous study on changes in urinary drug concentrations after the administration of heroin, cannabis, or cocaine followed by water intake in healthy adults, normal water intake did not significantly affect the drug-positive ratio of the participants when normalized by creatinine concentration. On the contrary, the creatinine concentration decreased and the drug-positive ratios of the participants increased due to the correction by creatinine concentration in excessively hydrated subjects (Cone et al., 2009). This approach could misestimate urinary drug or metabolite concentrations at the extremes of age and body size as creatinine excretion is determined by muscle mass (Dong et al., 2021), but it is widely used as a correction method for the quantitative results of drugs or metabolites in urine samples from healthy individuals (Barr et al., 2005). The weak correlation between unadjusted and creatinine-adjusted urinary GHB concentrations has been reported (Kang et al., 2014). As urinary volume is influenced by various factors such as kidney function, fluid intake, and perspiration (Carrieri et al., 2001), creatinine adjustment of the concentrations of GHB and its biomarker metabolites is recommended to compensate for individual variability in spot urine sampling.

It has been previously reported that creatinine is one of the human urinary metabolites that enables the classification of participants under and over 40 years old; the concentrations of creatinine in the former were higher than those in the latter (Slupsky et al., 2007). In the current study, neither the unadjusted nor the creatinine-adjusted concentrations of GHB were affected by differences in age. Serum metabolite profiling studies based on gender and age differences among the Japanese population have demonstrated that gender and age are key contributors clustering the subjects according to serum metabolites. Age-associated differences were observed in 24 and 23% of 516 endogenous serum metabolites in men and women, respectively, and the concentrations of glutamic acid and succinylcarnitine was higher in subjects aged 55–65 years as compared to subjects aged 25–35 years (Saito et al., 2016). Another metabolomics study had reported that the plasma concentrations of glutamic acid and succinic acid significantly increased and that of glycolic acid significantly decreased in older mice (72 weeks) as compared to those in young mice (8 weeks) (Seo et al., 2016a). The results of the present study are consistent with those in these two previous studies. Further, in a previous review on the aging metabolome, the central role of the TCA cycle in signaling and metabolic dysregulation associated with aging was emphasized (Sharma and Ramanathan, 2020).

Besides gender and age differences, a variety of intra-variable factors such as circadian rhythms (Slupsky et al., 2007; Bertram et al., 2009; Dallmann et al., 2012) and diet (van Duynhoven and Jacobs, 2016; Kessler and Pivovarova-Ramich, 2019) affect the human metabolome. In particular, a close relationship between the circadian clock and metabolic change has been demonstrated (Bellet and Sassone-Corsi, 2010; Masri and Sassone-Corsi, 2013; Davies et al., 2014). In the current study, significant inter-day variation of GABA concentration and intra-day variation of 3,4-OH-BA and succinylcarnitine concentrations were observed. A distinct time-of-day variation with a significant cosine fit during the wake/sleep cycle and during 24 h of wakefulness was observed in an untargeted and targeted analysis of two-hourly plasma samples in a previous study. Of the 171 metabolites quantified, daily rhythms were observed in the majority (*n* = 109), with 78 of these maintaining their rhythmicity during 24 h of wakefulness, most with reduced amplitude (*n* = 66) (Davies et al., 2014). It has been previously demonstrated that the metabolome of plasma and saliva is affected by the circadian clock; the concentrations of approximately 15% of all identified metabolites in plasma and saliva were altered during 40 h of extended wakefulness (Dallmann et al., 2012). In another study, the concentration of creatinine in urine collected during morning hours was significantly higher than that in the afternoon (Slupsky et al., 2007), which is consistent with our results.

As shown in Table 3, the urinary creatinine concentration increased significantly at 18:00–23:00 and 4:00–10:00 h. In previous human studies, elevated creatinine concentrations were observed in the morning, which could be because the urine is concentrated overnight (Barr et al., 2005; Slupsky et al., 2007). In another study, the creatinine concentrations were measured in urine collected from healthy adults at 9:30, 12:00, 14:30, 17:30, and 22:00 h, and the concentrations were higher at 9:30 and 22:00 h. The authors of the study found that the creatinine excretion rate in each individual was positively correlated with the urinary flow rate (Sallsten and Barregard, 2021). The creatinine concentrations in all urine samples in our study were acceptable for the criterion for screening drugs of abuse (≥ 5 mg/dl) set in the United States Department of Transportation Regulations (Barbanel et al., 2002). Therefore, the significant elevation in the creatinine concentration at 18:00–23:00 and 4:00–10:00 h was probably due to overnight urine concentration as well as decreased fluid intake and urine flow rate during evening and at night.

The biomarker metabolites quantified in the current study have been previously proposed as potential markers for GHB intoxication, but their correlation with GHB has not been investigated, which makes it difficult to utilize them in clinical and forensic practice. In this study, the urinary concentrations of 2,4-OH-BA, succinic acid, and 3,4-OH-BA showed the highest correlation with that of GHB. Succinic acid is not only one of the phase 1 metabolites of GHB but also an intermediate of the TCA cycle. Moreover, its role in pathological conditions such as inflammation and tumorigenesis has been emphasized (Mills and O'Neill, 2014; Tretter et al., 2016). Therefore, it appears to be unsuitable as a distinct marker for GHB intoxication. Nevertheless, the high correlation of 2,4-OH-BA or 3,4-OH-BA concentrations with those of GHB strongly supports their potential as direct markers of GHB intoxication.

In conclusion, this study provides data on urinary profiling of endogenous GHB and its biomarker metabolites in urine samples of 206 healthy females based on age and the time of sample collection. The data of the current study suggest population reference concentrations to facilitate clinical and forensic decisions associated with GHB-related intoxication and could prove useful in the verification of biomarker metabolites upon comparison with urinary profiles of GHB-administered populations.

## Data Availability

The original contributions presented in the study are included in the article/Supplementary Material, further inquiries can be directed to the corresponding authors.
