# Supplementary material for: Urinary Profile of Endogenous Gamma-Hydroxybutyric Acid and its Biomarker Metabolites in Healthy Korean Females: Determination of Age-Dependent and Intra-Individual Variability and Identification of Metabolites Correlated With Gamma-Hydroxybutyric Acid
Source: Front Pharmacol. 2022 Apr 13;13:853971. doi: 10.3389/fphar.2022.853971 (PMC9043528; doi:10.3389/fphar.2022.853971)
Supplement: Supplementary file 1 [file DataSheet1.docx]

Supplementary Material

# Supplementary Method

## Chemicals and reagents

All solvents were of high performance LC grade. GHB sodium salt, GHB-d_6_ sodium salt (1 mg/mL methanol solution for each), and Surine™ negative Urine Control were obtained from Cerilliant (Round Rock, TX, USA). 2,4-OH-BA lithium salt, 3,4-OH-BA lithium salt, glycolic acid, succinic acid, GABA, L-glutamic acid, O-succinyl-L-carnitine lithium salt, succinic acid-d_4_, GABA-d_6_, acetyl-L-carnitine-(N-methyl-d_3_) hydrochloride, sodium carbonate (anhydrous) and BzCl were purchased from Sigma-Aldrich (St. Louis, MO, USA). Isotope-labeled glycolic acid-d_2_ and L-glutamic acid-^13^C_5_D_5_^15^N were purchased from Cambridge Isotope Laboratories (Tewkesbury, MA, USA). All analytical stock solutions (1 mg/mL) except those of GHB or GHB-d_6_ were prepared in distilled water and stored at -80 °C. A working standard solution of GHB (2.48 μg/mL) as well as a working internal standard solution of GHB-d_6_ (3 μg/mL) were prepared in methanol. A working standard mixture solution of glutamic acid (15 μg/mL), GABA (3 μg/mL), succinic acid (30 μg/mL), 2,4-OH-BA (113 μg/mL), 3,4-OH-BA (99 μg/mL), glycolic acid (150 μg/mL) and succinylcarnitine (14.6 μg/mL) as well as a working internal standard mixture solution of glutamic acid-^13^C_5_D_5_^15^N (9 μg/mL), GABA-d_6_ (3 μg/mL), succinic acid-d_4_ (9 μg/mL), and acetylcarnitine-d_3_ (0.09 μg/mL) were prepared in distilled water. All the solutions were stored at -80 °C until further use.

## Quantification of GHB in urine samples (Method 1)

The urine samples were centrifuged at 14,000 rpm for 10 min at 4 °C to separate solid impurities followed by filtration of the respective supernatants using a 0.45 μm microporous polyvinylidene fluoride membrane. This was followed by addition of 10 µL each of the working internal standard solution of GHB-d_6_ to 20 µL each of the filtered urine samples followed by mixing for one minute. The mixture was centrifuged for five min and the respective supernatants were diluted twice with water. Finally, one µL each of the diluted supernatants was injected into the LC-MS/MS system. All experimental procedures were performed in an ice-bath.

An Acquity UPLC HSS T3 VanGuard Pre-Column (1.8 μm, Waters, Worchester, MA, USA) and an Acquity UPLC HSS T3 (2.1 mm × 100 mm, 1.8 μm, Waters), both maintained at 60 °C, were used as the guard column and the analytical column, respectively. Mobile phase A and B consisted of 0.1% formic acid in distilled water and 0.1% formic acid in 98% acetonitrile, respectively. The gradient conditions were as follows: 0-1.0 min, 0% B; 1.0-3.5 min, 0-20% B; 3.5-3.7 min, 20-80% B; 3.7-6.7 min, 80% B; 6.7-6.9 min, 80-0% B; 6.9-10 min, 0% B, at a flow rate of 250 μL/min. The temperature of the auto-sampler was set at 4 °C.

The MS/MS system was operated using the negative ion mode of electrospray ionization (ESI). Other conditions were optimized at the following values: capillary voltage, 3.5 kV; nebulization pressure, 60 psi; temperature of drying gas, 290 °C; drying gas flow, 14 L/min; sheath gas temperature, 400 °C; sheath gas flow, 11 L/min; nozzle voltage, 0.5 kV; and electron multiplier voltage, 0.4 kV. The analytes and internal standards were identified and quantified using multiple reaction monitoring (MRM). The MRM transitions, retention times and other conditions of GHB and GHB-d_6_ are listed in Supplementary Table 1.

## Quantification of glutamic acid, GABA, succinic acid, 2,4-OH-BA, 3,4-OH-BA, glycolic acid, and succinylcarnitine in urine samples (Method 2)

The urine samples were centrifuged and supernatants filtered as mentioned in Supplemental Method 2, section 2.1. Twenty microliters each of the filtered urine samples was mixed with 10 µL each of the respective internal standard solution. This was followed by addition of 10 µL each of 100 mM sodium carbonate buffer and BzCl (2% (v/v) in acetonitrile) solution to the samples for derivatization followed by mixing for one minute. The remaining steps in the procedure were as mentioned in the section 1.2.

The LC conditions were the same as those mentioned in Supplemental Method 2, section 2.2, except for the gradient conditions which were as follows: 0-1.0 min, 0% B; 1.0-3.0 min, 0-20% B; 3.0-5.5 min, 20% B; 5.5-9.5 min, 20-60% B; 9.5-9.6 min, 60% B; 9.6-12.5 min, 60% B (350 μL/min); 12.5-12.7 min, 60-0% B (350-300 μL/min); 12.7-14.5 min, 0% B (300-250 μL/min); 14.5-16 min, 0% B, at a flow rate of 250 μL/min, except for the flow rates specifically mentioned in parentheses.

The MS/MS system was operated using both the positive and negative ion modes of ESI. The other conditions were similar to those mentioned in Supplemental Method 2, section 2.2, except for capillary voltages for the positive and negative ion modes, which were 4.0 kV and 3.5 kV respectively. The MRM transitions, retention times and other conditions are listed in Supplementary Table 1.

## Validation study

The analytical methods were validated using synthetic urine as a blank matrix except for evaluation of matrix effects, for which human urine was used. The quality control (QC) samples were prepared by adding low (LQC) (Method 1: GHB, 0.025 μg/ mL; Method 2: glutamic acid, 0.3 μg/mL; GABA, 0.06 μg/mL; succinic acid, 0.6 μg/mL; 2,4-OH-BA, 2.3 μg/mL; 3,4-OH-BA, 2.0 μg/mL; glycolic acid, 7.5 μg/mL; succinylcarnitine, 0.29 μg/mL), medium (MQC) (Method 1: GHB, 0.33 μg/mL; Method 2: glutamic acid, 2 μg/mL; GABA, 0.4 μg/mL; succinic acid, 4 μg/mL; 2,4-OH-BA, 15 μg/mL; 3,4-OH-BA, 13 μg/mL; glycolic acid, 20 μg/mL; succinylcarnitine, 1.9 μg/mL), or high (HQC) (Method 1: GHB, 0.74 μg/mL; Method 2: glutamic acid, 4.5 μg/mL; GABA, 0.9 μg/mL; succinic acid, 9 μg/mL; 2,4-OH-BA, 34 μg/mL; 3,4-OH-BA, 30 μg/mL; glycolic acid, 45 μg/mL; succinylcarnitine, 4.4 μg/mL) concentrations of each analyte in the synthetic or human urine. Respective stable isotope-labeled compounds were used as the internal standards for each analyte, except for succinylcarnitine for which acetylcarnitine-d3 was used and glycolic acid, 2,4-OH-BA as well as 3,4-OH-BA for which GHB-d6 was used.

Evaluation of the matrix effects was conducted using three groups of internal standard-spiked samples; human urine samples collected from six individuals (Group A), human urine sample spiked with standards with the LQC and HQC levels (Group B), and neat solvent spiked with the LQC and HQC levels (Group C) and calculated as follows: matrix effects (%) = [(response ratio in Group B − response ratio in Group A) / response ratio in Group C] × 100 (1).

Other validation parameters of selectivity, sensitivity, linearity, precision, accuracy as well as stability were evaluated as described in previous studies (2, 3). To assess the selectivity as well as the sensitivity of the method, the blank synthetic urine sample, the synthetic urine sample spiked with the lower limit of quantification (LLOQ) levels of the analytes as well as the human urine sample were compared. Based on this, the absence of responses interfering with the signals of each analyte and internal standard in the synthetic urine as well as the method sensitivity for the detection of basal concentrations of the analytes was investigated. The analyte concentration at which the signal-to-noise ratio was > 3 was chosen as the limit of detection (LOD) whereas the analyte concentration with < 20% coefficient of variation (CV) for precision and < ±20% for bias, was selected for the LLOQ. Three sets of calibration curves were prepared using the synthetic urine and analyzed to demonstrate linearity. Method precision and accuracy were examined using the synthetic samples spiked with the concentration levels of LLOQ, LQC, MQC, and HQC of each analyte. Quintuple samples for each concentration were prepared and analyzed on three separate days. Following storage for 24 h, three replicates of the LQC as well as HQC samples were reinjected in the autosampler at 4°C for the evaluation of processed sample stability.

# Supplementary Results

## Validation study

During sample preparation of the target compounds (GHB, glutamic acid, GABA, succinic acid, 2,4-OH-BA, 3,4-OH-BA, glycolic acid, and succinylcarnitine), benzoylation was used to increase the sensitivity as well as selectivity of glutamic acid and GABA. However, it aggravated the sensitivity of GHB; therefore, Method 1 (without benzoylation) was used for the quantification of GHB exclusively and Method 2 (with benzoylation) for that of other metabolites.

Both methods were successfully validated (Supplementary Figure 1 and Supplementary Table 2). No interference was observed in the retention times in synthetic urine. The endogenous concentrations of the target compounds were distinctly detected in the authentic human urine. The results of other method validation parameters are summarized in Supplemental Table 2. In Method 1, the LOD and LLOQ values of GHB were 0.004 and 0.008 μg/mL, respectively. No significant matrix effects were observed with a coefficient of variation (CV) of 12.3% and 3.3% in LQC and HQC, respectively. Further, the method proved to be precise (less than 11.6% of CV) and accurate (less than 5.8% of bias) with acceptable linearity (r=0.9992) within calibration ranges (0.008-0.8 μg/mL). The stability of GHB after the process was satisfactory with no tendency of degradation (95.4% and 95.3% in LQC and HQC, respectively). In Method 2, the LOD and LLOQ values were between 0.005 μg/mL (Bz-GABA) and 2.5 μg/mL (glycolic acid) and between 0.02 μg/mL (Bz-GABA) and 5 μg/mL (glycolic acid), respectively. Acceptable linearity was created within each calibration range, with the ‘r’ value of each analyte ranging from 0.9954 (glycolic acid) to 0.9987 (succinylcarnitine). The mean values of the matrix effect of each analyte at low and high concentrations ranged from 82.6% (glycolic acid in HQC) to 233.2% (succinylcarnitine in LQC), respectively. Minor ion suppression or enhancement was observed with a coefficient of variation (CV) less than 20% (range: 4.0%–17.0%). The CVs of repeatability and intermediate precision ranged from 3.5% (2,4-OH-BA) to 11.9% (Bz-glutamic acid) and 4.1% (3,4-OH-BA) to 11.3% (succinylcarnitine), respectively, and were within acceptable limits (i.e., < ± 20% CV at low concentrations and < ±15% CV at medium as well as high concentrations, respectively). The accuracy of this method ranged from a bias of –12.8% (succinylcarnitine) to 10.0% (succinic acid) at the three concentration levels and was within acceptable limits (i.e., < ± 20% bias at low concentrations and ± 15% bias at medium as well as high concentrations). Process stability was also satisfactory ranging from 90.0% (Bz-glutamic acid) to 105.5% (succinylcarnitine). Therefore, the entire urinary profiling process was performed under the current optimized conditions.

**Supplementary Table 1.** MRM transitions, retention times and other conditions for each analyte and internal standard

|  | Compound name | Precursor  ion (*m/z*) | Product  ion (*m/z*) | tR (min) | Frag (V) | CE (V) | Ionization polarity |
| --- | --- | --- | --- | --- | --- | --- | --- |
| Method 1 | GHB | 102.9 | 85.0  101.0 | 2.4 | 380 | 8  8 | Negative |
|  | GHB-d_6_ | 109.1 | 89.9 | 2.4 | 380 | 16 | Negative |
| Method 2 | Bz-GABA | 208.0 | 86.0 | 6.1 | 380 | 14 | Positive |
|  |  |  | 105.1 |  |  | 33 |  |
|  | Bz-Glutamic acid | 252.0 | 105.0 | 5.3 | 380 | 33 | Positive |
|  |  |  | 130.0 |  |  | 8 |  |
|  | Succinylcarnitine | 262.3 | 60.2 | 2.2 | 380 | 16 | Positive |
|  |  |  | 84.9 |  |  | 28 |  |
|  | Succinic acid | 116.8 | 73.2  98.9 | 2.6 | 380 | 8  8 | Negative |
|  | Glycolic acid | 75.1 | 47.2 | 1.3 | 380 | 8 | Negative |
|  | 2,4-OH-BA | 119.0 | 43.2  55.1 | 1.5 | 380 | 12  18 | Negative |
|  | 3,4-OH-BA | 119.0 | 59.2 | 1.5 | 380 | 8 | Negative |
|  |  |  |  |  |  |  |  |
|  | Bz-GABA-d_6_ | 214.0 | 77.1 | 6.1 | 380 | 49 | Positive |
|  | Bz-Glutamic acid-^13^C_5_D_5_^15^N | 263.0 | 141.0 | 5.3 | 380 | 8 | Positive |
|  | Acetylcarnitine-d_3_ | 206.9 | 85.1 | 1.8 | 380 | 21 | Positive |
|  | Succinic acid-d_4_ | 121.1 | 102.0 | 2.5 | 380 | 8 | Negative |

Underlined transitions were used for quantification.

GHB, Gamma-hydroxybutyric acid; Bz, Benzoylated; GABA, Gamma-aminobutyric acid; 2,4-OH-BA, 2,4-dihydroxybutyric acid; 3,4-OH-BA, 3,4-dihydroxybutyric acid; tR, retention time; Frag, fragmentation voltage; CE, collision energy.

**Supplementary Table 2.** MRM transitions, retention times and other conditions for each analyte and internal standard

| Compound name | LOD (μg/mL) | LLOQ (μg/mL) | Calibration range (μg/mL) | Regression coefficient (r, mean) | Spiked concentration level | Matrix effect (%) | | Repeatability^a^ (CV, %) | Intermediate precision^b^ (CV, %) | Accuracy (Bias, %) | Processed sample stability |
| --- | --- | --- | --- | --- | --- | --- | --- | --- | --- | --- | --- |
|  |  |  |  |  |  | Mean | CV |  |  |  |  |
| Method 1 | | | | | | | | | | | |
| GHB | 0.0041 | 0.0083 | 0.0083-0.83 | 0.9992 | LLOQ | - | - | 8.5 | 11.6 | 5.8 | - |
|  |  |  |  |  | LQC | 97.8 | 12.3 | 6.8 | 8.3 | 2.1 | 95.4 |
|  |  |  |  |  | MQC | - | - | 4.4 | 6.6 | 3.1 | - |
|  |  |  |  |  | HQC | 101.3 | 3.3 | 3.2 | 8.9 | 2.2 | 95.3 |
| Method 2 | | | | | | | | | | | |
| Bz-GABA | 0.005 | 0.02 | 0.02-1 | 0.9963 | LLOQ | - | - | 7.2 | 8.5 | -4.8 | - |
|  |  |  |  |  | LQC | 97.4 | 12.3 | 4.2 | 7.4 | -3.5 | 97.3 |
|  |  |  |  |  | MQC | - | - | 10.9 | 8.7 | 5.5 | - |
|  |  |  |  |  | HQC | 96.8 | 7.6 | 8.1 | 8.8 | 2.6 | 94.2 |
| Bz-Glutamic acid | 0.025 | 0.1 | 0.1-5 | 0.9983 | LLOQ | - | - | 7.0 | 9.0 | -4.9 | - |
|  |  |  |  |  | LQC | 92.5 | 11.4 | 11.9 | 7.6 | -6.9 | 98.8 |
|  |  |  |  |  | MQC | - | - | 5.0 | 8.5 | 1.5 | - |
|  |  |  |  |  | HQC | 108.4 | 6.0 | 7.3 | 8.9 | 3.1 | 90.0 |
| Succinylcarnitine | 0.024 | 0.097 | 0.097-4.9 | 0.9987 | LLOQ | - | - | 4.2 | 10.8 | -12.8 | - |
|  |  |  |  |  | LQC | 233.2 | 17.0 | 6.1 | 11.3 | 2.9 | 105.5 |
|  |  |  |  |  | MQC | - | - | 6.2 | 8.0 | 6.5 | - |
|  |  |  |  |  | HQC | 166.3 | 12.6 | 7.9 | 6.2 | -0.3 | 97.0 |
| Succinic acid | 0.05 | 0.2 | 0.2-10 | 0.9968 | LLOQ | - | - | 4.8 | 8.3 | 10.0 | - |
|  |  |  |  |  | LQC | 90.1 | 6.7 | 9.2 | 9.0 | -7.6 | 96.6 |
|  |  |  |  |  | MQC | - | - | 5.4 | 5.6 | -4.6 | - |
|  |  |  |  |  | HQC | 99.9 | 4.0 | 6.3 | 6.0 | -0.8 | 99.9 |
| Glycolic acid | 2.5 | 5 | 5-50 | 0.9954 | LLOQ | - | - | 9.6 | 8.8 | -0.3 | - |
|  |  |  |  |  | LQC | 87.0 | 14.3 | 5.2 | 9.1 | 0.0 | 101.7 |
|  |  |  |  |  | MQC | - | - | 5.6 | 5.0 | 3.5 | - |
|  |  |  |  |  | HQC | 82.6 | 13.4 | 6.7 | 6.1 | 0.4 | 101.5 |
| 2,4-OH-BA | 0.19 | 0.76 | 0.76-38 | 0.9979 | LLOQ | - | - | 4.2 | 7.8 | -1.9 | - |
|  |  |  |  |  | LQC | 94.8 | 6.3 | 4.0 | 5.5 | -7.6 | 97.1 |
|  |  |  |  |  | MQC | - | - | 5.2 | 4.2 | 5.5 | - |
|  |  |  |  |  | HQC | 98.4 | 12.3 | 3.5 | 3.9 | -1.0 | 96.8 |
| 3,4-OH-BA | 0.17 | 0.66 | 0.66-33 | 0.9963 | LLOQ | - | - | 6.6 | 10.6 | -4.3 | - |
|  |  |  |  |  | LQC | 84.1 | 13.6 | 5.1 | 4.7 | -1.8 | 103.3 |
|  |  |  |  |  | MQC | - | - | 3.6 | 4.1 | 6.5 | - |
|  |  |  |  |  | HQC | 93.3 | 12.7 | 5.1 | 4.3 | -5.0 | 97.0 |

GHB, Gamma-hydroxybutyric acid; Bz, Benzoylated; GABA, Gamma-aminobutyric acid; 2,4-OH-BA, 2,4-dihydroxybutyric acid; 3,4-OH-BA, 3,4-dihydroxybutyric acid; LOD, limit of detection; LLOQ, lower limit of quantification; LQC, low concentration-quality control sample; MQC, medium concentration-quality control sample; HQC, high concentration-quality control sample CV, coefficient of variation.

^a^Within-day variation.

^b^Combination of within- and between-day variation

**Supplementary Table 3. Unadjusted and creatinine-adjusted concentrations of endogenous GHB and its biomarker metabolites by ages**

| Compound name | Age (years) | Number of samples | Unadjusted concentrations (μg/mL) | | | | Creatinine-adjusted concentrations (μg/mg creatinine) | | | |
| --- | --- | --- | --- | --- | --- | --- | --- | --- | --- | --- |
|  |  |  | Mean | Median | Range | SD | Mean | Median | Range | SD |
| GHB | 20–29 | 155 | 0.11 | 0.08 | 0.01-0.59 | 0.10 | 0.09 | 0.08 | 0.02-0.32 | 0.05 |
|  | 30-39 | 153 | 0.11 | 0.07 | 0.01-0.75 | 0.12 | 0.11 | 0.08 | 0.03-0.64 | 0.10 |
|  | 40-49 | 152 | 0.09 | 0.06 | 0.01-0.48 | 0.08 | 0.11 | 0.08 | 0.03-1.3 | 0.13 |
| Glutamic acid | 20–29 | 158 | 1.3 | 0.92 | 0.16-8.0 | 1.2 | 1.3 | 1.1 | 0.47-4.7 | 0.58 |
|  | 30-39 | 156 | 1.3 | 0.96 | 0.11-11 | 1.3 | 1.3 | 1.2 | 0.42-5.5 | 0.55 |
|  | 40-49 | 157 | 1.4 | 0.87 | 0.14-13 | 1.7 | 1.7 | 1.4 | 0.33-11 | 1.2 |
| GABA | 20–29 | 147 | 0.15 | 0.12 | 0.02-1.4 | 0.15 | 0.13 | 0.12 | 0.04-0.81 | 0.09 |
|  | 30-39 | 143 | 0.13 | 0.10 | 0.02-0.67 | 0.11 | 0.14 | 0.12 | 0.03-0.65 | 0.08 |
|  | 40-49 | 149 | 0.11 | 0.08 | 0.02-0.49 | 0.08 | 0.14 | 0.13 | 0.01-0.50 | 0.06 |
| Succinic acid | 20–29 | 138 | 1.1 | 0.82 | 0.21-6.5 | 1.0 | 0.92 | 0.81 | 0.29-3.5 | 0.55 |
|  | 30-39 | 126 | 1.2 | 0.68 | 0.21-12 | 1.5 | 1.1 | 0.76 | 0.21-12 | 1.3 |
|  | 40-49 | 122 | 1.1 | 0.77 | 0.20-5.2 | 1.0 | 1.2 | 0.92 | 0.27-11 | 1.3 |
| 2,4-OH-BA | 20–29 | 143 | 5.6 | 3.9 | 0.84-36 | 5.6 | 4.3 | 3.7 | 1.7-13 | 2.0 |
|  | 30-39 | 140 | 4.6 | 3.3 | 0.78-21 | 4.4 | 4.3 | 3.9 | 1.3-13 | 2.2 |
|  | 40-49 | 146 | 3.6 | 2.4 | 0.78-26 | 3.4 | 4.2 | 3.6 | 1.8-22 | 2.2 |
| 3,4- OH-BA | 20–29 | 156 | 7.9 | 5.5 | 0.66-53 | 8.0 | 6.4 | 5.9 | 2.4-23 | 2.8 |
|  | 30-39 | 156 | 6.6 | 4.2 | 0.69-34 | 6.3 | 6.7 | 6.2 | 1.8-25 | 3.3 |
|  | 40-49 | 154 | 5.5 | 3.9 | 0.66-26 | 4.6 | 6.7 | 6.3 | 2.8-25 | 3.2 |
| Glycolic acid | 20–29 | 143 | 29 | 22 | 5.1-207 | 27 | 25 | 21 | 5.2-143 | 18 |
|  | 30-39 | 135 | 24 | 17 | 5.1-181 | 23 | 24 | 21 | 6.4-162 | 19 |
|  | 40-49 | 126 | 18 | 14 | 5.1-63 | 13 | 20 | 18/ | 3.5-55 | 8.9 |
| Succinylcarnitine | 20–29 | 131 | 0.77 | 0.55 | 0.10-2.6 | 0.62 | 0.52 | 0.50 | 0.15-1.2 | 0.19 |
|  | 30-39 | 122 | 0.71 | 0.53 | 0.10-2.9 | 0.63 | 0.54 | 0.53 | 0.13-12 | 0.19 |
|  | 40-49 | 130 | 0.68 | 0.42 | 0.10-4.3 | 0.70 | 0.60 | 0.55 | 0.21-1.5 | 0.23 |

The values less than the limit of quantification were excluded. SD, standard deviation; GHB, Gamma-hydroxybutyric acid; GABA, Gamma-aminobutyric acid; 2,4-OH-BA, 2,4-dihydroxybutyric acid; 3,4-OH-BA, 3,4-dihydroxybutyric acid

**Supplementary Table 4. Unadjusted and creatinine-adjusted concentrations of endogenous GHB and its biomarker metabolites by different sampling days**

| Compound name | Sampling day | Number of samples | Unadjusted concentrations (μg/mL) | | | | Creatinine-adjusted concentrations (μg/mg creatinine) | | | |
| --- | --- | --- | --- | --- | --- | --- | --- | --- | --- | --- |
|  |  |  | Mean | Median | Range | SD | Mean | Median | Range | SD |
| GHB | Day 1 | 174 | 0.11 | 0.07 | 0.01-0.59 | 0.11 | 0.11 | 0.08 | 0.02-1.3 | 0.12 |
|  | Day 2 | 168 | 0.10 | 0.08 | 0.01-0.46 | 0.09 | 0.10 | 0.08 | 0.02-0.64 | 0.09 |
| Glutamic acid | Day 1 | 179 | 1.2 | 0.85 | 0.14-8.0 | 1.1 | 1.4 | 1.2 | 0.33-11 | 0.96 |
|  | Day 2 | 173 | 1.5 | 1.1 | 0.12-13 | 1.6 | 1.4 | 1.3 | 0.48-5.5 | 0.85 |
| GABA | Day 1 | 170 | 0.13 | 0.10 | 0.02-0.77 | 0.11 | 0.14 | 0.12 | 0.01-0.65 | 0.08 |
|  | Day 2 | 162 | 0.13 | 0.11 | 0.02-0.67 | 0.10 | 0.13 | 0.12 | 0.03-0.46 | 0.06 |
| Succinic acid | Day 1 | 156 | 1.2 | 0.76 | 0.21-12 | 1.4 | 1.2 | 0.86 | 0.26-12 | 1.3 |
|  | Day 2 | 148 | 1.2 | 0.81 | 0.20-6.5 | 1.0 | 1.1 | 0.86 | 0.21-11 | 1.0 |
| 2,4-OH-BA | Day 1 | 168 | 4.6 | 3.1 | 0.82-23 | 4.4 | 4.4 | 3.9 | 1.5-22 | 2.3 |
|  | Day 2 | 161 | 4.7 | 3.3 | 0.78-26 | 4.7 | 4.2 | 3.7 | 1.3-13 | 2.2 |
| 3,4- OH-BA | Day 1 | 178 | 6.6 | 4.5 | 0.69-37 | 6.2 | 6.8 | 5.9 | 1.8-23 | 3.5 |
|  | Day 2 | 173 | 6.9 | 4.6 | 0.66-53 | 7.0 | 6.5 | 6.1 | 1.8-25 | 3.3 |
| Glycolic acid | Day 1 | 158 | 24 | 18 | 5.1-181 | 24 | 24 | 20 | 5.2-146 | 18 |
|  | Day 2 | 149 | 23 | 17 | 5.1-108 | 17 | 22 | 19 | 5.5-162 | 15 |
| Succinylcarnitine | Day 1 | 144 | 0.73 | 0.52 | 0.10-2.9 | 0.62 | 0.57 | 0.52 | 0.15-1.5 | 0.21 |
|  | Day 2 | 146 | 0.73 | 0.50 | 0.10-2.8 | 0.61 | 0.55 | 0.54 | 0.13-1.0 | 0.20 |

The values less than the limit of quantification were excluded. SD, standard deviation; GHB, Gamma-hydroxybutyric acid; GABA, Gamma-aminobutyric acid; 2,4-OH-BA, 2,4-dihydroxybutyric acid; 3,4-OH-BA, 3,4-dihydroxybutyric acid

**Supplementary Table 5. Unadjusted and creatinine-adjusted concentrations of endogenous GHB and its biomarker metabolites by different sampling times**

| Compound name | Sampling time | Number of samples | Unadjusted concentrations (μg/mL) | | | | Creatinine-adjusted concentrations (μg/mg creatinine) | | | |
| --- | --- | --- | --- | --- | --- | --- | --- | --- | --- | --- |
|  |  |  | Mean | Median | Range | SD | Mean | Median | Range | SD |
| GHB | 18:00-23:00 | 30 | 0.15 | 0.11 | 0.01-0.75 | 0.15 | 0.12 | 0.10 | 0.05-0.47 | 0.08 |
|  | 04:00-10:00 | 30 | 0.13 | 0.08 | 0.02-0.71 | 0.15 | 0.09 | 0.06 | 0.03-0.35 | 0.06 |
|  | 10:00-14:00 | 28 | 0.06 | 0.03 | 0.01-0.38 | 0.08 | 0.08 | 0.07 | 0.04-0.22 | 0.04 |
|  | 14:00-18:00 | 30 | 0.03 | 0.02 | 0.01-0.13 | 0.03 | 0.08 | 0.07 | 0.03-0.24 | 0.04 |
| Glutamic acid | 18:00-23:00 | 30 | 1.7 | 1.2 | 0.11-10 | 1.8 | 1.3 | 1.1 | 0.59-3.9 | 0.72 |
|  | 04:00-10:00 | 30 | 1.9 | 1.6 | 0.52-4.9 | 1.3 | 1.4 | 1.3 | 0.83-2.6 | 0.42 |
|  | 10:00-14:00 | 29 | 0.92 | 0.62 | 0.13-4.5 | 0.93 | 1.4 | 1.4 | 0.71-2.4 | 0.44 |
|  | 14:00-18:00 | 30 | 0.57 | 0.49 | 0.12-3.3 | 0.57 | 1.5 | 1.3 | 0.83-4.7 | 0.70 |
| GABA | 18:00-23:00 | 29 | 0.18 | 0.14 | 0.05-1.4 | 0.24 | 0.16 | 0.11 | 0.04-0.81 | 0.16 |
|  | 04:00-10:00 | 30 | 0.16 | 0.13 | 0.05-0.54 | 0.11 | 0.12 | 0.13 | 0.04-0.27 | 0.05 |
|  | 10:00-14:00 | 24 | 0.09 | 0.07 | 0.03-0.53 | 0.10 | 0.14 | 0.13 | 0.04-0.26 | 0.06 |
|  | 14:00-18:00 | 24 | 0.06 | 0.05 | 0.02-0.21 | 0.04 | 0.16 | 0.17 | 0.04-0.28 | 0.06 |
| Succinic acid | 18:00-23:00 | 25 | 1.0 | 0.70 | 0.24-3.4 | 0.80 | 0.73 | 0.65 | 0.28-1.8 | 0.38 |
|  | 04:00-10:00 | 29 | 1.2 | 1.1 | 0.23-4.7 | 1.0 | 0.81 | 0.72 | 0.26-2.3 | 0.47 |
|  | 10:00-14:00 | 18 | 0.68 | 0.48 | 0.23-2.0 | 0.51 | 0.82 | 0.70 | 0.38-1.9 | 0.38 |
|  | 14:00-18:00 | 10 | 0.53 | 0.35 | 0.21-1.6 | 0.47 | 1.0 | 0.86 | 0.29-2.0 | 0.57 |
| 2,4-OH-BA | 18:00-23:00 | 28 | 6.0 | 5.0 | 1.1-21 | 4.2 | 4.6 | 4.4 | 1.9-13 | 2.2 |
|  | 04:00-10:00 | 30 | 6.3 | 4.4 | 1.3-36 | 6.7 | 4.2 | 4.0 | 1.8-11 | 2.0 |
|  | 10:00-14:00 | 23 | 2.8 | 1.7 | 0.82-10 | 2.3 | 3.5 | 3.4 | 2.1-6.8 | 1.1 |
|  | 14:00-18:00 | 19 | 1.6 | 1.4 | 0.78-5.6 | 1.1 | 3.4 | 3.4 | 1.7-5.6 | 0.97 |
| 3,4- OH-BA | 18:00-23:00 | 29 | 9.1 | 8.6 | 0.99-22 | 5.4 | 7.2 | 6.9 | 4.2-13 | 2.2 |
|  | 04:00-10:00 | 30 | 10 | 6.9 | 2.6-42 | 8.5 | 7.0 | 6.1 | 4.2-14 | 2.5 |
|  | 10:00-14:00 | 26 | 4.3 | 2.7 | 0.89-16 | 3.5 | 5.9 | 5.8 | 3.6-9.8 | 1.5 |
|  | 14:00-18:00 | 30 | 2.1 | 1.6 | 0.71-8.9 | 1.6 | 5.7 | 5.4 | 3.0-9.3 | 1.6 |
| Glycolic acid | 18:00-23:00 | 27 | 31 | 27 | 6.3-65 | 17 | 24 | 21 | 4.0-41 | 10 |
|  | 04:00-10:00 | 30 | 33 | 17 | 6.9-207 | 41 | 24 | 18 | 3.5-125 | 23 |
|  | 10:00-14:00 | 21 | 17 | 13 | 5.1-49 | 12 | 24 | 22 | 8.1-69 | 12 |
|  | 14:00-18:00 | 19 | 11 | 8.0 | 5.1-42 | 9.2 | 25 | 23 | 11-59 | 12 |
| Succinylcarnitine | 18:00-23:00 | 28 | 0.95 | 0.64 | 0.11-3.4 | 0.85 | 0.63 | 0.58 | 0.31-1.3 | 0.24 |
|  | 04:00-10:00 | 30 | 0.87 | 0.62 | 0.10-4.3 | 0.87 | 0.57 | 0.53 | 0.28-1.5 | 0.24 |
|  | 10:00-14:00 | 19 | 0.42 | 0.27 | 0.10-1.3 | 0.37 | 0.44 | 0.42 | 0.20-0.91 | 0.17 |
|  | 14:00-18:00 | 16 | 0.23 | 0.21 | 0.10-0.76 | 0.15 | 0.44 | 0.45 | 0.21-0.64 | 0.12 |

The values less than the limit of quantification were excluded. SD, standard deviation; GHB, Gamma-hydroxybutyric acid; GABA, Gamma-aminobutyric acid; 2,4-OH-BA, 2,4-dihydroxybutyric acid; 3,4-OH-BA, 3,4-dihydroxybutyric acid

**
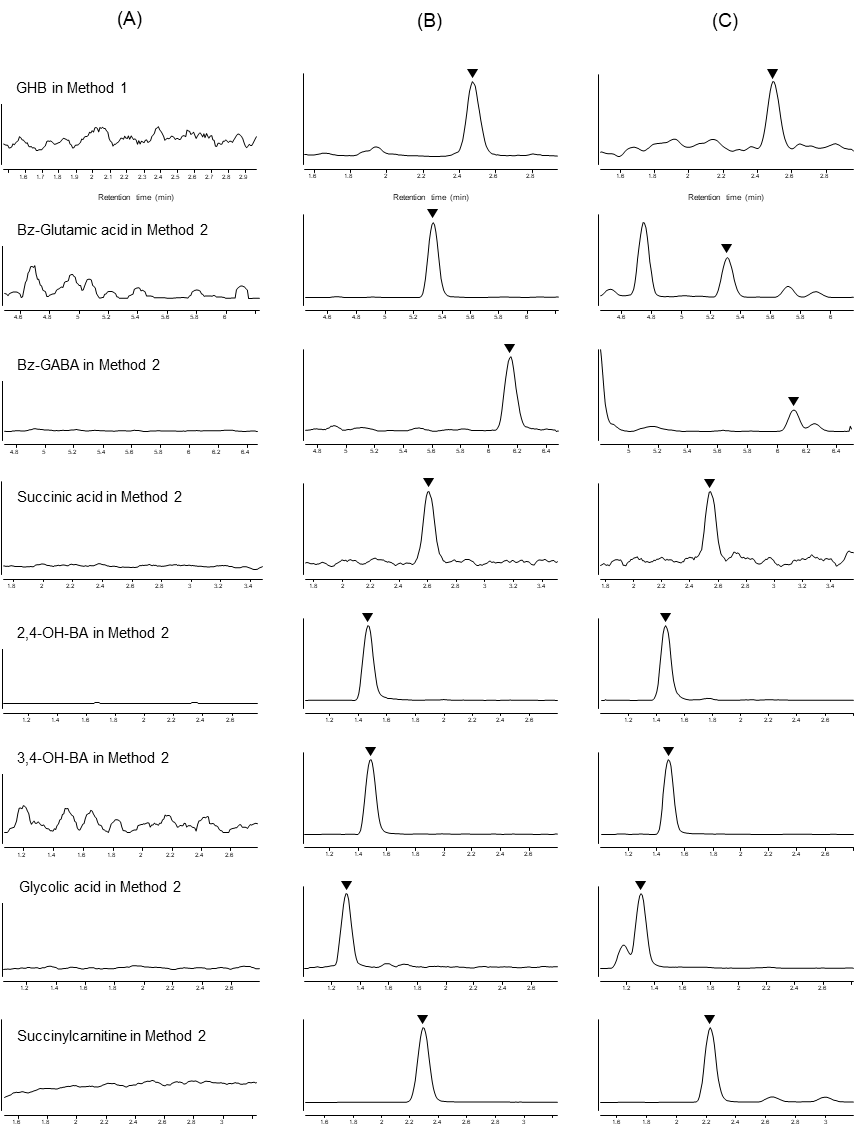
**

**Supplementary Figure 1.** Representative chromatograms of the quantifier mass transitions of target compounds using Method 1 (without benzoylation) or Method 2 (with benzoylation) [(A) synthetic urine; (B) fortified synthetic urine (LLOQ level); (C) a human urine sample]

**References**

1. Khamis MM, Adamko DJ, El-Aneed A. Strategies and Challenges in Method Development and Validation for the Absolute Quantification of Endogenous Biomarker Metabolites Using Liquid Chromatography-Tandem Mass Spectrometry. Mass Spectrom Rev 2021;40:31-52.

2. Peters FT, Drummer OH, Musshoff F. Validation of new methods. Forensic Sci Int 2007;165:216-24.

3. Peters FT. Method validation. In: Polettini A, eds. Applications of LC-MS in Toxicology. London: Pharmaceutical Press, 2006:71-95.
